# Brain anatomy differences in Chinese children who stutter: a preliminary study

**DOI:** 10.3389/fneur.2025.1483157

**Published:** 2025-01-27

**Authors:** Dan Ma, Lingling Wang, Sai Liu, XinMao Ma, Fenglin Jia, Yimin Hua, Yi Liao, Haibo Qu

**Affiliations:** ^1^Department of Rehabilitation Medicine, West China Second University Hospital, Sichuan University, Chengdu, Sichuan, China; ^2^Key Laboratory of Birth Defects and Related Diseases of Women and Children (Sichuan University), Ministry of Education, Chengdu, Sichuan, China; ^3^Department of Radiology, West China Second University Hospital, Sichuan University, Chengdu, China; ^4^Department of Pediatrics, West China Second University Hospital, Sichuan University, Chengdu, Sichuan, China

**Keywords:** developmental stuttering, gray matter, cortical morphology, structural magnetic resonance, Chinese children

## Abstract

**Background and purpose:**

It is unknown the neural mechanisms of developmental stuttering (DS). The aim of this study was to investigate the changes in the structural morphology of the brain in Chinese children who stutter.

**Methods:**

A case–control study was conducted to collect magnetic resonance imaging data from stuttering and non-stuttering children, thereby analyzing whole-brain gray matter volume and cortical morphological changes in stuttering children.

**Results:**

A total of 108 subjects were recruited (stuttering group: control group = 1:1). Comparing to healthy controls, the gray matter volume was significantly decreased in right temporal gyrus and bilateral cerebellum. Additionally, there was a significant reduction in cortical folds in the right insula and right superior temporal gyrus. Moreover, the gray matter volume of the right cerebellum and right temporal gyrus is related to the severity score of stuttering.

**Conclusion:**

The present study proposes that the neural mechanisms underlying DS are intricately linked to the cortico-basal ganglia-thalamo-cortical loop and the dorsal language pathway. This finding is expected to provide reference value for the clinical treatment of DS.

## Introduction

Developmental stuttering (DS) is a childhood-onset speech fluency disorder that occurs most often in children between the ages of 2 and 5 years (1, 2). About 5–10% of preschool children have been reported to stutter, and 75–80% of these children recover spontaneously within 2–3 years (3). Persistent stuttering can greatly reduce the overall quality of life of children and adolescents, limiting their participation in activities, affecting their academic performance, socialization, etc. (4–6). By identifying the structural brain abnormalities involved in stuttering helps to explore effective treatments (7).

There is increasing evidence for differences in Morphological Structure of the Brain in Children Who Stutter. Stuttering is a neuromotor disorder involving neural networks and sensorimotor areas involved in motor programming and control, like damaged/abnormal cortical white and gray matter structures, altered neural activity in the cortico-basal-thalamic-cortical circuits (7). These “neural markers” may alter sensorimotor interactions at a neural level (8). Jäncke et al. (9) first used the voxel based morphometry (VBM) method to study gray matter volume changes in adults who stutter, and he found anatomical abnormalities in the lateral perisylvian fissure area, prefrontal lobe, and sensorimotor areas in stutterers. Using the same method for the first time in 2007, the Chinese team found a significant increase in gray matter volume in the superior temporal gyrus, middle temporal gyrus, postcentral gyrus, inferior parietal lobule and premotor gyrus of the bilateral cerebral hemispheres, and a significant decrease in gray matter volume in the posterior cerebellar lobes and medulla oblongata in the stuttering group (10). Neuroimaging studies on children who stutter have found that children with a history of stuttering have less gray matter volume in the left inferior frontal gyrus and supplementary motor areas (11). To further study the cortical morphology of stuttering, the surface based morphometry (SBM) has been gradually applied. Related studies point to increased cortical folding in stuttering (9, 12). Garnett et al. Analyzed the brain morphology of children who stuttered by the SBM method and found that children who stuttered had reduced cortical thickness in the left ventral motor cortex and ventral premotor cortex regions (13, 14).

Current research on the brain structure and function of children who stutter has made great progress (15). However, there are some shortcomings. Chinese children have differences in language structure, expression, and family upbringing, which can influence the existence of differences in brain structure in different language environment (16–18). Moreover, China has a large population base, and there is a great market demand for early diagnosis and accurate treatment for children who stutter and their families. However, there are fewer studies related to brain structure and function in Chinese children who stutter. Secondly, most of the studies used a single VBM method, or SBM method, focusing on localized or specific types of information. Finally, although many studies have shown that DS is associated with neuroanatomical abnormalities in speech-producing neural networks (15). However, the results reported in the literature are inconsistent when it comes to the location and orientation of the brain regions with differences. This is mainly related to inconsistent study populations, sample size differences and incomplete research techniques.

In summary, the present study was conducted to reveal the brain morphology and structural differences in Chinese children who stutter by means of structural magnetic resonance and to investigate the relationship between the structural brain alterations and clinical features of DS.

## Methods

### Patients selection

Participants were enrolled from February 2019 to November 2020, 54 pediatric patients with stuttering were enrolled. Fifty-four healthy pediatric controls of who are recruited for the study criteria for both sex and age were enrolled in the research. The present research was authorized from the Ethics Committee. All pediatric patients’ legal guardians gave written informed consent. The criteria for the diagnosis of stuttering (Childhood-Onset Fluency Disorder) were developed based on the Diagnostic and Statistical Manual of Mental Disorders-5 (DSM-V) (1, 19). The criteria for the Stuttering in DSM-V are as below: (A) Disturbances in the temporal pattern and normal fluency of speech that are incompatible with the age and speech ability of the individual, long duration, and with the characteristics of marked and frequent occurrence of one (or more) of the following: (i) Repetition of sounds and syllables. (ii) Prolongation of the sound of vowels and consonants. (iii) Word breaks (for example, a pause within a word). (iv) Audible or non-audible obstructions (filled or unfilled pauses in speech). (v) Circumlocution (a word substitution is intended to avoid a problematic word). (vi) Words generated in a situation of excessive physical tension. (vii) A full word repetition of a single syllable. (B) The disorder results in individuals experiencing anxiety when speaking, or limited in the communication with people, academics, social participation as well as the occupational performance, alone or in any combination. (C) Symptoms appear in the early stages of development (Note: Cases of late onset are diagnosed as a fluency disorder of adult onset). (D) The disorder cannot be ascribed to a speech motor or sensory deficit, a fluency disorder related to a neurological injury (such as trauma, tumor, and stroke), or other clinical condition, and cannot be better interpreted with another psychiatric disorder.

All children included in the experiment were required to meet the following inclusion criteria: (1) native Chinese language, (2) age ≤ 14 years, (3) right-handedness, and (4) no organic brain parenchymal lesions suggested by imaging. The exclusion criteria for children in the stuttering group were as shown below: (1) oral malformations such as cleft lip and palate, (2) hearing abnormality, (3) intellectual disability, autism spectrum disorders, cerebral palsy, and other disorders leading to speech disorders, (4) psychiatric disorders and medication-induced stuttering manifestations, (5) MRI scans showing signal or structural abnormalities in intracranial lesions, (6) patients with claustrophobia or other conditions that preclude MRI scans, and (7) patients with a history of psychiatric disorders. The exclusion criteria for the control group were as follows: (1) psychiatric, neurological or other metabolic disorders, (2) MRI scans showing signal or structural abnormalities in intracranial lesions, (3) patients with claustrophobia or other conditions that preclude MRI scans, and (4) patients with a history of psychiatric disorders.

Moreover, participation was conditional on the absence of language or speech impairment and mental retardation on standardized tests by both the Wechsler Intelligence Scale for Children, Fourth Edition (WISC-IV) and the Diagnostic Receptive and Expressive Assessment of Mandarin-Comprehensive (DREAM-C). Children who scored 1 standard deviation below the average of the standardized measures above were not allowed to participate. The severity of stuttering for participation was evaluated with Stuttering Severity Instrument-Version 4 (SSI-4).

### Structural magnetic resonance imaging acquisition

A standardized process of clinician evaluation and collection was employed for the collection of clinical data.

All of the participants received high-resolution anatomical whole brain T1-weighted MRI in a 3.0 Tesla Siemens Skyra scanner. The parameters of the 3D T1 sequence are listed below: repetition time of 5 ms, echo time of 2.95 ms, matrix dimensions of 192 × 192, field of view of 200 mm × 200 mm, flip angle of 0°, voxel dimensions of 1.04 mm × 1.04 mm × 1.00 mm, and 144 axial slices. During scanning, the participants were instructed to maintain immobility of the head while in a state of relaxation with their eyes closed. Earplugs and foam padding were used to diminish ambient noise and restrict head movement. To eliminate the influence of head motion, participants with a maximum displacement of >3 mm and maximum rotation of >3° were excluded from further analysis.

Prior to analysis, all of the structural images were evaluated by three experienced researchers. Images affected by motion artifacts were excluded. In this study, 2 stuttering participants and 3 typical development participants were excluded.

## MRI processing

### VBM

T1-weighted structural MRI images of participants were analyzed with Statistical Parametric Software (SPM12^1^) by adopting the default parameters. The major processing steps include: (i) manually re-orienting the images, (ii) extracting the brains, and which were segmented into maps of cerebrospinal fluid (CSF), white matter (WM) as well as gray matter (GM), (iii) normalizing all the above images to the Montreal Neurological Institute (MNI) space, (iv) smoothing with full-width at half-maximum (FWHM) Gaussian kernel of 8 mm. Statistical analyses were subsequently performed on the smoothed images to investigate voxel-wise differences of GM volumes between the stuttering and typical development groups. To avoid involving WM portion, voxels with GM intensities below a threshold of 0.1 (maximum GM intensity was 1) were excluded. Thereafter a two-sample t-test was conducted on the intensity plot of GM and total intracranial volume (TIV), sex and age were added as covariates. Statistical significance thresholds were set at a combination of cluster-level *p* < 0.05 and voxel-level *p* < 0.001 for multi-comparisons with the method of Gaussian Random Field (GRF).

### SBM

Surface based morphometry analysis was performed using CAT12 (Computational Anatomy Toolbox^2^) software. The main steps included: (i) the individual spatial T1W was radiometrically aligned to MNI152 space, and non-brain tissues were excluded, (ii) the white matter tissue signal intensity change was estimated, and the field estimation was performed on the whole brain based on the white matter tissue body, (iii) the white matter distribution in the standard brain tissue template of MNI152, and the signal intensity and spatial location of somatotropin in the individual space were used to analyze the white matter tissue body that might belong to the white matter tissue in the T1W image of the individual, (iv) generation of an initial sphere in each of the left and right hemispheres to convert from voxel-based volume space to vertex-based surface space analysis. The construction of white matter-gray matter and gray matter-cerebrospinal fluid boundary models can be realized by intensity gradient detection, respectively, and the distance between the two boundaries is the gray matter cortical thickness, (v) Gaussian smoothing kernel FWHM = 15 mm was chosen for cortical thickness index, and Gaussian smoothing kernel FWHM = 20 mm was chosen for the other indexes, (vi) Based on the measured distance, we calculated the cortical thickness, cortical surface area, mean curvature and sulcus depth to analyze the changes of brain surface morphology.

### Statistical analysis

Statistical analyses were performed using SPSS version 23 (IBM Corp., Armonk, NY, United States). Continuous variables were expressed as mean ± standard deviation, while categorical data were expressed as counts and percentages. A 2-tailed *p* < 0.05 was considered statistically significant differences. We analyzed the data and Demographic characterization data conformed to normal distribution and variance chi-square. VBM data belonged to skewed distribution and non-parametric test was used. SBM data belonged to normal distribution and two-sample *t*-test was used. Images from the two groups were compared in terms of gray matter and cortical morphology, adjusting for age and gender as covariates. Each pixel’s value was computed to generate a statistical parameter map. Pseudo-colored displays were used to represent *t*-values exceeding the threshold, with the darkness of the color spectrum indicating the magnitude of the *t*-value. Subsequently, the biased correlation between the brain structural eigenvalues of these regions and stuttering severity scores in the patient group was calculated, adjusting for factors such as age, gender, and total intracranial volume.

## Results

### Baseline characteristics

A total of 54 cases in the stuttering group and 54 cases in the healthy control group were included in this study, there was no statistically significant difference in age and gender between the healthy control group and the stuttering group (*p* > 0.05) (Table 1).

**Table 1:** Baseline characteristics.

	Stuttering group, *n* = 54	Control group, *n* = 54	*p*-value
Age (years)	7.01 ± 2.509	6.95 ± 2.593	0.91
Sex, *n* (%)			0.62
Boy	43 (79.6)	45 (83.3)	
Girl	11 (20.4)	9 (16.7)	
IQ			
Full Scale IQ	102.69 ± 10.363	/	NA
Verbal Comprehension Index	104.65 ± 12.083	/	NA
Perceptual Reasoning Index	NA	/	NA
Fluid Reasoning Index	NA	/	NA
Working Memory Index	98.43 ± 9.822	/	NA
Processing Speed Index	96.07 ± 10.634	/	NA
Visual Spatial Index	NA	/	NA
Stuttering Severity Instrument	23.91 ± 6.585	/	NA

IQ, Intelligenzquotient; NA, not applicable.

### Data results based on VBM analysis

**Figure 1:**
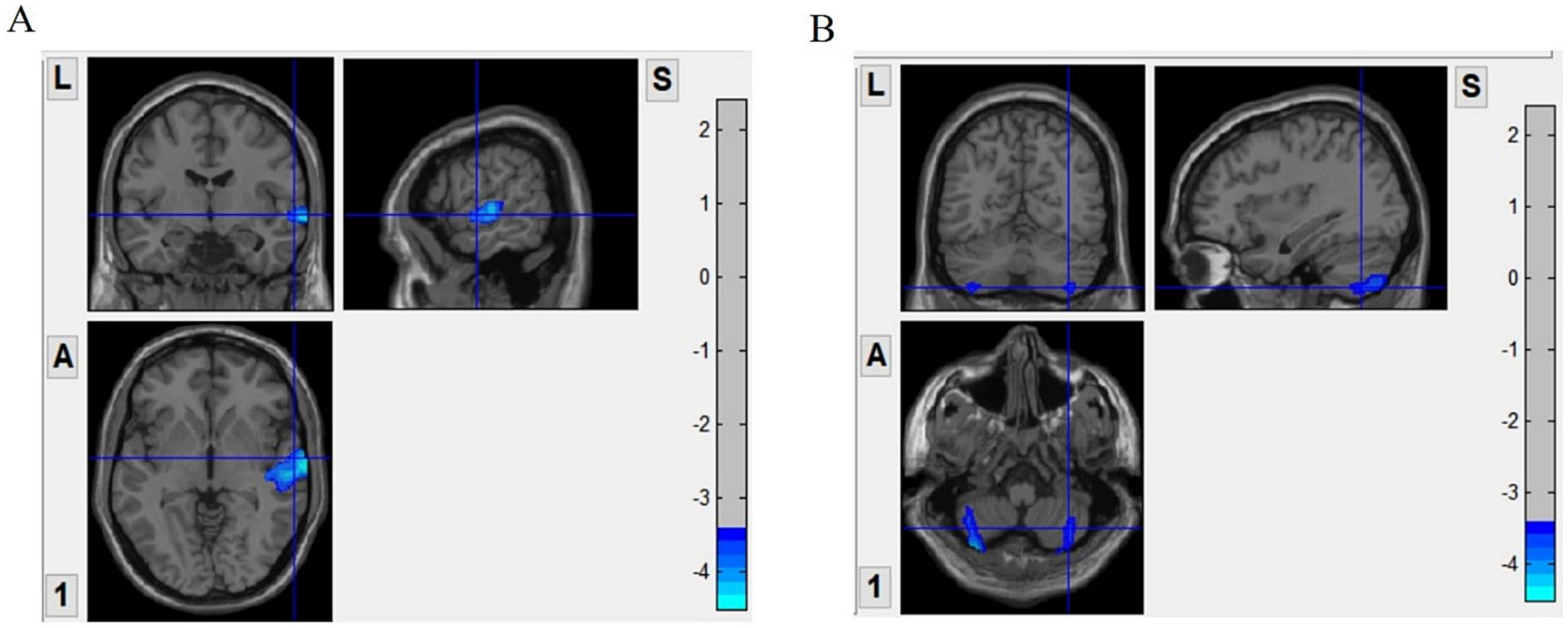
Surface based morphometry (SBM)-based analysis of brain regions with significant differences in gray matter volume between the two groups. Panel **(A)** shows that the stuttering group had significantly less gray matter volume in the right temporal gyrus compared to the control group. Panel **(B)** shows a significant reduction in gray matter volume in the bilateral cerebellum of the stuttering group. VBM, voxel-based morphometry.

The gray matter volumes of the stuttering and control groups were statistically analyzed using non-parametric tests, and age, gender, and total intracranial volume were used as covariates to compare the differences between the two groups. There were significant differences in gray matter volumes in the right temporal gyrus and bilateral cerebellum in patients who stuttered. Gray matter volume was significantly reduced in the right temporal gyrus and bilateral cerebellum (Figure 1) compared to healthy controls (Table 2 and Figure 1). In addition, correlation analysis showed that stuttering severity scores were positively correlated with gray matter volume in the right temporal gyrus (*r* = 0.412, *p* = 0.002) and right cerebellar gray matter volume (*r* = 0.289, *p* = 0.034), respectively.

**Table 2:** Data results based on VBM analysis.

Brain area	Cluster size (mm^2^)	MNI coordinates			Stuttering group	Control group	*p*-value
		*X*	*Y*	*Z*			
Left cerebellum	1,419	−36	−82.5	−48	0.489 (95% CI, 0.445–0.520)	0.502 (95% CI, 0.470–0.556)	0.044
Right cerebellum	635	30	−81	−52.5	0.590 (95% CI, 0.544–0.629)	0.612 (95% CI, 0.564–0.673)	0.024
Right temporal gyrus	1963	70.5	−7.5	−4.5	0.546 (95% CI, 0.499–0.596)	0.595 (95% CI, 0.547–0.632)	<0.001

VBM, voxel-based morphometry.

### Data results based on SBM analysis

**Figure 2:**
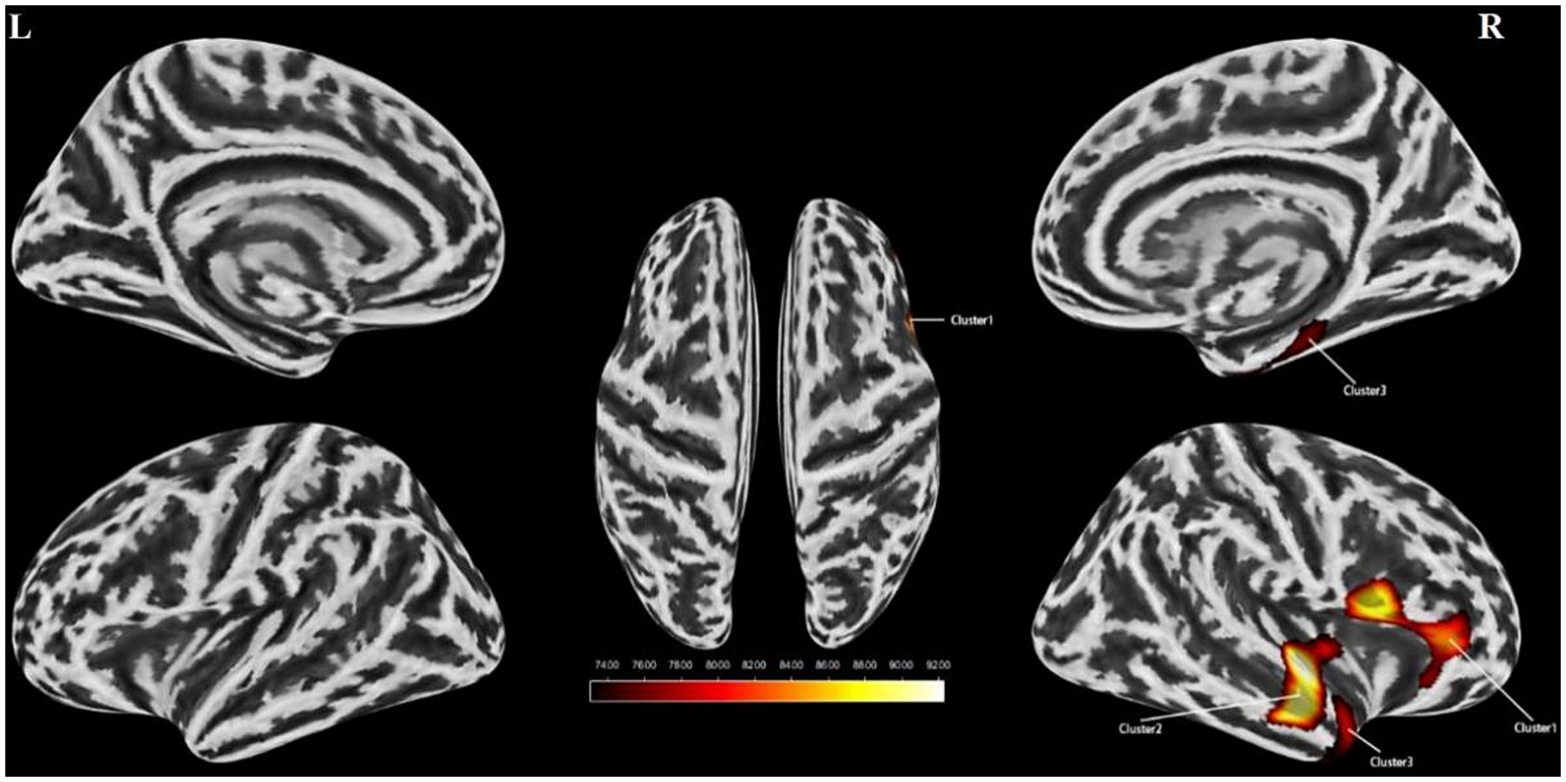
Surface based morphometry-based analysis of the brain regions where the two groups differed significantly in cortical folds. Clusters with reduced cortical folds in the stuttering group compared to the control group (yellow clusters) Cluster 1 represents the right insula, Cluster 2 represents the right superior temporal gyrus, and Cluster 3 represents the right superior temporal gyrus. l, left hemisphere; r, right hemisphere. Color bands indicate *t*-values (red indicates that cortical folds were smaller in the stuttering group than in the healthy control group). SBM, Surface-based Morphometry.

An independent t-test was conducted to assess cortical thickness, sulcus depth, fractal dimension, and cortical fold characteristics of the stuttering group versus the control group. Multiple corrections were applied to compare the statistical outcomes. Our findings indicated a substantial decrease in cortical folds specifically in the right insula and the right superior temporal gyrus among individuals with stuttering (Table 3 and Figure 2). However, no significant disparities were observed between the two groups in terms of cortical surface area, sulcus depth, or fractal dimension. Furthermore, a partial correlation analysis was performed between cortical fold metrics and stuttering severity scores among the stuttering group. These results revealed no significant correlation between the three brain regions and stuttering severity scores (*P* > 0.05).

**Table 3:** Data results based on SBM analysis.

Brain area	Cluster size (mm^2^)	*P*-value
Right insula	729	0.025
Right superior temporal gyrus	667	0.021
Right superior temporal gyrus	591	0.037

SBM, surface-based Morphometry.

## Discussion

To the best of our knowledge, this study took Chinese children who stutter as subjects to explore morphological and structural brain differences in this population. The main findings of this study were a significantly decreased in right temporal gyrus and bilateral cerebellum in the gray matter volume; a significant reduction in cortical folds in the right insula and right superior temporal gyrus.

The posterior superior temporal gyrus (pSTG) has been reported to be interconnected with the premotor and subfrontal areas through numerous primary white matter tracts (20). Speech preparation and execution in stutterers cannot be isolated from sensory areas, since speech movements are scheduled and performed as a consequence of speech movements against the background of the intended sensory goal (19). There is much evidence for abnormal interactions between the speech auditory-motor integration of person who stutters (22). As is well known, the manipulations of auditory feedback (such as masking noise, delayed auditory feedback or frequency-shifted feedback) can at least temporarily fluency the stutterer (23–25). Research on auditory interference with sensory-motor adaptation has demonstrated that stuttering adults show decreased adaptive ability in comparison with controls, while stuttering children present the same adaptive ability as controls. Notably, speech-induced auditory cortical inhibition was delayed in children who stuttered at latency, suggesting that even in the children, there is an interaction between auditory and the motor areas (26, 27). Inactivation of the left auditory cortex (STG, middle temporal gyrus) in the speech task in stutterers is among the ‘neural features’ emphasized in the meta-analysis of PET and fMRI investigations of stuttering (28, 29). Of interest is the fact that bilateral right STG and pSTG activity is particularly enhanced in people who stutter when they speak fluently (30). Fluency conditions induced, like rhythmic choral singing and speech by a metronome, revealed increased auditory cortical activity compared to reading alone, exceeding that of controls in the same areas (31). While this is speculative, it may suggest that when stuttering is expected, auditory cortex deactivation occurs to minimize the perceived mismatch between the predicted and actual speech (32). Our study also found that children who stutter have decreased gray matter volume in the right temporal gyrus, which is generally consistent with those of previous studies (33). Unfortunately, the reason for the decreased gray matter volume in the right superior temporal gyrus in patients who stutter is unknown. It has been suggested that the change of gray matter volume in the right superior temporal gyrus may be a compensatory structural change due to a speech motor disorder (32, 34).

Besides, changes in cortical morphology of the brain are also strongly associated with stuttering. The Gyrification Index (GI) serves as a quantitative measure of the intricate surface patterns of the cerebral cortex (35). The insula, a crucial component of Broca’s area, covers the cortex and plays a pivotal role in numerous brain functions (36). It maintains close ties with regions like Broca’s area, the frontal lobe, parietal lobe, and motor cortex (37). Meanwhile, the superior temporal gyrus, a vital part of Wernicke’s area, is responsible for processing auditory information. This gyrus connects with the insula via the dorsal auditory pathway, which is crucial for speech perception, maintaining phonological working memory for perceived information, and speech production (both articulatory planning and execution) (38). Studies have hinted at structural or functional irregularities in the insula and superior temporal gyrus among those who stutter, such as reduced volume and intensified brain activity (14, 39). Our research echoes these findings, revealing a significant decrease in cortical folds within the right insula and right superior temporal gyrus among stutterers. This reduction suggests potential disruptions in neuronal connections between the right insula and the right superior temporal gyrus, potentially affecting the dorsal auditory pathway’s role in speech motor processes. However, the present study found no significant correlation between the three abnormal brain regions and stuttering severity scores. Possible Reasons Heterogeneity of stuttering behavior and changes in brain morphology among stutterers are not caused by just one brain region or a single factor, but are the result of the interaction of multiple brain regions and factors. The relationship between structural brain changes and the assessment of stuttering symptoms still requires further research to deepen the understanding of the mechanisms underlying stuttering.

Several studies have pointed to the involvement of the cerebellum in the coordination of speech-related movements (8, 40). Kotz et al. (41) and Schwartze et al. (42) have proposed a valuable subcortical framework for the perception and production of speech sounds. Here, it is believed that the interactions between auditory cortical areas and temporal processing systems (cerebellum and basal ganglia) built fundamental timing routines in the acquisition of speech. In such framework, once routines have been acquired, the contribution of the auditory-cerebellar-thalamic-frontal-striatal (BG) is considered a complementary function, while the cerebellum remains involved actively in computing sensory information. Based on the work of Stockert et al. (43) and Pinheiro et al. (44), the audiological information can be transmitted to the temporal processing system of the cerebellum through the neural pathway between the cochlear nucleus and cerebellum. The cerebellum projects in sequence through the thalamus to the frontal cortex; the frontal cortex is then attached to the BG, creating a circuit around BG region (44). In addition, the basal ganglia is a key site for the initiation of speech motor programs that are associated with the pathogenesis of DS (45). The basal ganglia-cerebellum-cerebral cortex network can provide part of the basis for the executive control network, which is associated with executive function, working memory, and verbal fluency (46). Some studies have found that in people who stutter. Abnormal activity occurs in the basal ganglia, and to compensate for this abnormal activity, the cerebellum becomes more active, and it is hypothesized that the cerebellum may provide a means of compensating for impaired basal ganglia function in stuttering patients (47). The gray matter volume results of the present study showed a significant reduction in bilateral cerebellar gray matter volume in the stuttering group. This affects the effective connection between the cerebellum and the basal ganglia, leading to a loss of compensation in the brain’s coordinated speech motor mechanisms. This is likely to be one of the reasons for the development of stuttering. Our findings reaffirm the importance of the cerebellum in stuttering-related brain regions.

This study is based on VBM and SBM analyses, which effectively combine local and global brain structural features in people who stutter. The cerebellum has extensive connections to the cerebral cortex and subcortical regions, with the cerebellum projecting to the striatum through dense synapses in the dentate nucleus and interconnecting with the subcortical basal ganglia to form a complete network with the cerebral cortex (38, 46). The basal ganglia-cerebellum-cerebral cortex network can provide part of the basis for the executive control network, which is associated with verbal fluency (38, 46). Our findings show a reduction in cortical folds in the right insula of the cerebellar gray matter body and the right superior temporal gyrus in patients who stutter, which may result in slowed or interrupted information transfer between different brain regions in the brain structure of patients who stutter, leading to instability in language planning and control, which may manifest itself in symptoms of stuttering such as syllable repetition or syllable lengthening. The results of this study provide reference value for clinicians and therapists in the diagnosis and treatment of stuttering disorder. First, early diagnosis and prediction of stuttering disorder is expected to be realized through structural changes in the cerebellum and temporal gyrus. Secondly, it is also possible to select appropriate targets through central nervous stimulation to promote structural changes in the brain associated with DS, and thus improve stuttering symptoms. Finally, effective stuttering treatments can also be sought through these structural brain changes, providing evidence-based medical evidence for the treatment of stuttering.

## Limitations

This study possesses several constraints. Firstly, this leads to the fact that the external validity of the results of this study is not yet known and it is more difficult to apply the results of a single center sample to other regions and populations. A larger sample from multiple centers must be taken to confirm the reliability of our findings. Secondly, this is a cross-sectional study which does not allow us to understand the systematic and continuous changes in DS. Therefore, further research is necessary to elucidate the association between stuttering and temporal changes in cortical structures and subcortical areas of the brain. Finally, changes in brain function also have an impact on stuttering, and abnormal functional connectivity in the amygdala, chiasma, and pons may lead to stuttering. This study is based on structural magnetic resonance as a research method, which is not yet able to understand the performance of active brain regions and the functional connectivity and synergy between different brain regions in children with stuttering in recognizing a specific task. In the future, we will study brain network changes in children with DS during resting and task states to explore the functional brain characteristics of DS.

## Conclusion

The present study revealed that abnormalities in cerebellar and superior temporal gyrus gray matter volumes and cortical folds were associated with children with DS. These structural alterations in brain regions involve brain areas and pathways associated with speech motor control, particularly involving the cortico-basal ganglia-thalamo-cortical loop and the dorsal language pathway.

## Data Availability

The original contributions presented in the study are included in the article/supplementary material, further inquiries can be directed to the corresponding authors.

## Footnotes

^1^ https://www.fil.ion.ucl.ac.uk/spm/software/spm12/

^2^ https://neuro-jena.github.io/cat//index.html#DOWNLOAD

## References

[1] First MB. Diagnostic and statistical manual of mental disorders, 5th edition, and clinical utility. J Nerv Ment Dis. (2013) 201:727–9. doi: 10.1097/NMD.0b013e3182a2168a, PMID: 23995026

[2] Neumann K Euler HA Bosshardt HG Cook S Sandrieser P Sommer M. The pathogenesis, assessment and treatment of speech fluency disorders. Dtsch Arztebl Int. (2017) 114:383–90. doi: 10.3238/arztebl.2017.0383, PMID: 28655373 PMC5504509

[3] Koenraads SPC van der Schroeff MP van Ingen G Lamballais S Tiemeier H Baatenburg de Jong RJ. Structural brain differences in pre-adolescents who persist in and recover from stuttering. Neuroimage Clin. (2020) 27:102334. doi: 10.1016/j.nicl.2020.102334, PMID: 32650280 PMC7341447

[4] McAllister J. Behavioural, emotional and social development of children who stutter. J Fluen Disord. (2016) 50:23–32. doi: 10.1016/j.jfludis.2016.09.003, PMID: 27865227

[5] Bernard R Hofslundsengen H Frazier Norbury C. Anxiety and depression symptoms in children and adolescents who stutter: a systematic review and Meta-analysis. J Speech Lang Hear Res. (2022) 65:624–44. doi: 10.1044/2021_JSLHR-21-00236, PMID: 35084999

[6] Erickson S Block S. The social and communication impact of stuttering on adolescents and their families. J Fluen Disord. (2013) 38:311–24. doi: 10.1016/j.jfludis.2013.09.003, PMID: 24331240

[7] Chang SE Angstadt M Chow HM Etchell AC Garnett EO Choo AL. Anomalous network architecture of the resting brain in children who stutter. J Fluen Disord. (2018) 55:46–67. doi: 10.1016/j.jfludis.2017.01.002, PMID: 28214015 PMC5526749

[8] Neef NE Anwander A Friederici AD. The neurobiological grounding of persistent stuttering: from structure to function. Curr Neurol Neurosci Rep. (2015) 15:63. doi: 10.1007/s11910-015-0579-4, PMID: 26228377

[9] Jäncke L Hänggi J Steinmetz H. Morphological brain differences between adult stutterers and non-stutterers. BMC Neurol. (2004) 4:23. doi: 10.1186/1471-2377-4-23, PMID: 15588309 PMC539354

[10] Song LP Peng DL Jin Z Yao L Ning N Guo XJ. Gray matter abnormalities in developmental stuttering determined with voxel-based morphometry. Zhonghua Yi Xue Za Zhi. (2007) 87:2884–8. doi: 10.3760/j.issn:0376-2491.2007.41.002 PMID: 18261300

[11] Koenraads SPC el Marroun H Muetzel RL Chang SE Vernooij MW Baatenburg de Jong RJ. Stuttering and gray matter morphometry: a population-based neuroimaging study in young children. Brain Lang. (2019) 194:121–31. doi: 10.1016/j.bandl.2019.04.008 31085031

[12] Cykowski MD Kochunov PV Ingham RJ Ingham JC Mangin JF Riviere D. Perisylvian sulcal morphology and cerebral asymmetry patterns in adults who stutter. Cereb Cortex. (2008) 18:571–83. doi: 10.1093/cercor/bhm093, PMID: 17584852

[13] Garnett EO Chow HM Nieto-Castañón A Tourville JA Guenther FH Chang SE. Anomalous morphology in left hemisphere motor and premotor cortex of children who stutter. Brain. (2018) 141:2670–84. doi: 10.1093/brain/awy199, PMID: 30084910 PMC6113637

[14] Garnett EO Chow HM Chang SE. Neuroanatomical correlates of childhood stuttering: MRI indices of white and gray matter development that differentiate persistence versus recovery. J Speech Lang Hear Res. (2019) 62:2986–98. doi: 10.1044/2019_JSLHR-S-CSMC7-18-0356, PMID: 31465710 PMC6813035

[15] Etchell AC Civier O Ballard KJ Sowman PF. A systematic literature review of neuroimaging research on developmental stuttering between 1995 and 2016. J Fluen Disord. (2018) 55:6–45. doi: 10.1016/j.jfludis.2017.03.007, PMID: 28778745

[16] Wang X Yang J Yang J Mencl WE Shu H Zevin JD. Language differences in the brain network for reading in naturalistic story reading and lexical decision. PLoS One. (2015) 10:e0124388. doi: 10.1371/journal.pone.0124388, PMID: 26017384 PMC4446262

[17] Zhu S Chong S Chen Y Wang T Ng ML. Effect of language on voice quality: an acoustic study of bilingual speakers of mandarin Chinese and English. Folia Phoniatr Logop. (2022) 74:421–30. doi: 10.1159/000525649, PMID: 35764052 PMC9808749

[18] Hu W Lee HL Zhang Q Liu T Geng LB Seghier ML. Developmental dyslexia in Chinese and English populations: dissociating the effect of dyslexia from language differences. Brain. (2010) 133:1694–706. doi: 10.1093/brain/awq106, PMID: 20488886 PMC2877905

[19] American Psychiatric Association. Diagnostic and statistical manual of mental disorders. Text revision. (2000).

[20] Micheli C Schepers IM Ozker M Yoshor D Beauchamp MS Rieger JW. Electrocorticography reveals continuous auditory and visual speech tracking in temporal and occipital cortex. Eur J Neurosci. (2020) 51:1364–76. doi: 10.1111/ejn.13992, PMID: 29888819 PMC6289876

[21] Craig-McQuaide A Akram H Zrinzo L Tripoliti E. A review of brain circuitries involved in stuttering. Front Hum Neurosci. (2014) 8:884. doi: 10.3389/fnhum.2014.00884 25452719 PMC4233907

[22] Kim KS Max L. Speech auditory-motor adaptation to formant-shifted feedback lacks an explicit component: reduced adaptation in adults who stutter reflects limitations in implicit sensorimotor learning. Eur J Neurosci. (2021) 53:3093–108. doi: 10.1111/ejn.15175, PMID: 33675539 PMC8259784

[23] Assaneo MF Ripollés P Tichenor SE Yaruss JS Jackson ES. The relationship between auditory-motor integration, interoceptive awareness, and self-reported stuttering severity. Front Integr Neurosci. (2022) 16:869571. doi: 10.3389/fnint.2022.869571, PMID: 35600224 PMC9120354

[24] Tumanova V Zebrowski PM Goodman SS Arenas RM. Motor practice effects and sensorimotor integration in adults who stutter: evidence from visuomotor tracking performance. J Fluen Disord. (2015) 45:52–72. doi: 10.1016/j.jfludis.2015.04.001, PMID: 25990027 PMC4546883

[25] Frankford SA Cai S Nieto-Castañón A Guenther FH. Auditory feedback control in adults who stutter during metronome-paced speech II. Formant Perturbation J Fluency Disord. (2022) 74:105928. doi: 10.1016/j.jfludis.2022.105928, PMID: 36063640 PMC9930613

[26] Daliri A Wieland EA Cai S Guenther FH Chang SE. Auditory-motor adaptation is reduced in adults who stutter but not in children who stutter. Dev Sci. (2018) 21:2521. doi: 10.1111/desc.12521, PMID: 28256029 PMC5581739

[27] Cai S Beal DS Ghosh SS Guenther FH Perkell JS. Impaired timing adjustments in response to time-varying auditory perturbation during connected speech production in persons who stutter. Brain Lang. (2014) 129:24–9. doi: 10.1016/j.bandl.2014.01.002, PMID: 24486601 PMC3947674

[28] Beal DS Gracco VL Lafaille SJ de Nil LF. Voxel-based morphometry of auditory and speech-related cortex in stutterers. Neuroreport. (2007) 18:1257–60. doi: 10.1097/WNR.0b013e3282202c4d, PMID: 17632278

[29] Salmelin R Schnitzler A Schmitz F Jäncke L Witte OW Freund HJ. Functional organization of the auditory cortex is different in stutterers and fluent speakers. Neuroreport. (1998) 9:2225–9. doi: 10.1097/00001756-199807130-00014, PMID: 9694204

[30] Mock JR Foundas AL Golob EJ. Cortical activity during cued picture naming predicts individual differences in stuttering frequency. Clin Neurophysiol. (2016) 127:3093–101. doi: 10.1016/j.clinph.2016.06.005, PMID: 27472545 PMC5053619

[31] Hampton A Weber-Fox C. Non-linguistic auditory processing in stuttering: evidence from behavior and event-related brain potentials. J Fluen Disord. (2008) 33:253–73. doi: 10.1016/j.jfludis.2008.08.001, PMID: 19328979 PMC2663969

[32] Watkins KE Smith SM Davis S Howell P. Structural and functional abnormalities of the motor system in developmental stuttering. Brain. (2008) 131:50–9. doi: 10.1093/brain/awm241, PMID: 17928317 PMC2492392

[33] Beal DS Gracco VL Brettschneider J Kroll RM de Nil LF. A voxel-based morphometry (VBM) analysis of regional grey and white matter volume abnormalities within the speech production network of children who stutter. Cortex. (2013) 49:2151–61. doi: 10.1016/j.cortex.2012.08.013, PMID: 23140891 PMC3617061

[34] Ismail N Sallam Y Behery R al Boghdady A. Cortical auditory evoked potentials in children who stutter. Int J Pediatr Otorhinolaryngol. (2017) 97:93–101. doi: 10.1016/j.ijporl.2017.03.030, PMID: 28483259

[35] Zilles K Palomero-Gallagher N Amunts K. Development of cortical folding during evolution and ontogeny. Trends Neurosci. (2013) 36:275–84. doi: 10.1016/j.tins.2013.01.006, PMID: 23415112

[36] Chang SE Horwitz B Ostuni J Reynolds R Ludlow CL. Evidence of left inferior frontal-premotor structural and functional connectivity deficits in adults who stutter. Cereb Cortex. (2011) 21:2507–18. doi: 10.1093/cercor/bhr028, PMID: 21471556 PMC3183422

[37] Fedorenko E Blank IA. Broca’s area is not a natural kind. Trends Cogn Sci. (2020) 24:270–84. doi: 10.1016/j.tics.2020.01.001, PMID: 32160565 PMC7211504

[38] Brauer J Anwander A Perani D Friederici AD. Dorsal and ventral pathways in language development. Brain Lang. (2013) 127:289–95. doi: 10.1016/j.bandl.2013.03.001, PMID: 23643035

[39] Beal DS Lerch JP Cameron B Henderson R Gracco VL De Nil LF. The trajectory of gray matter development in Broca's area is abnormal in people who stutter. Front Hum Neurosci. (2015) 9:89. doi: 10.3389/fnhum.2015.00089 25784869 PMC4347452

[40] Lu C Long Y Zheng L Shi G Liu L Ding G. Relationship between speech production and perception in people who stutter. Front Hum Neurosci. (2016) 10:224. 27242487 10.3389/fnhum.2016.00224 PMC4870257

[41] Kotz SA D’Ausilio A Raettig T Begliomini C Craighero L Fabbri-Destro M. Lexicality drives audio-motor transformations in Broca’s area. Brain Lang. (2010) 112:3–11. doi: 10.1016/j.bandl.2009.07.008, PMID: 19698980

[42] Schwartze M Tavano A Schröger E Kotz SA. Temporal aspects of prediction in audition: cortical and subcortical neural mechanisms. Int J Psychophysiol. (2012) 83:200–7. doi: 10.1016/j.ijpsycho.2011.11.003, PMID: 22108539

[43] Stockert A Schwartze M Poeppel D Anwander A Kotz SA. Temporo-cerebellar connectivity underlies timing constraints in audition. eLife. (2021) 10:10. doi: 10.7554/eLife.67303, PMID: 34542407 PMC8480974

[44] Pinheiro AP Schwartze M Kotz SA. Cerebellar circuitry and auditory verbal hallucinations: an integrative synthesis and perspective. Neurosci Biobehav Rev. (2020) 118:485–503. doi: 10.1016/j.neubiorev.2020.08.004, PMID: 32810512

[45] Busan P. Developmental stuttering and the role of the supplementary motor cortex. J Fluen Disord. (2020) 64:105763. doi: 10.1016/j.jfludis.2020.105763, PMID: 32361030

[46] Bostan AC Strick PL. The basal ganglia and the cerebellum: nodes in an integrated network. Nat Rev Neurosci. (2018) 19:338–50. doi: 10.1038/s41583-018-0002-7, PMID: 29643480 PMC6503669

[47] Bostan AC Dum RP Strick PL. Functional anatomy of basal ganglia circuits with the cerebral cortex and the cerebellum. Prog Neurol Surg. (2018) 33:50–61. doi: 10.1159/000480748, PMID: 29332073

